# Difference in Frequency and Distribution of Nodal Metastases Between Intermediate and High Risk Prostate Cancer Patients: Results of a Superextended Pelvic Lymph Node Dissection

**DOI:** 10.3389/fsurg.2018.00052

**Published:** 2018-09-07

**Authors:** Marco Roscigno, Maria Nicolai, Giovanni La Croce, Federico Pellucchi, Manuela Scarcello, Antonino Saccà, Diego Angiolilli, Daniela Chinaglia, Luigi F. Da Pozzo

**Affiliations:** ^1^Department of Urology, ASST Papa Giovanni XXIII, Bergamo, Italy; ^2^FROM Research Foundation, ASST Papa Giovanni XXIII, Bergamo, Italy; ^3^Department of Pathology, ASST Papa Giovanni XXIII, Bergamo, Italy

**Keywords:** prostate cancer, lymph node dissection, lymph node template, staging, nodal metastases

## Abstract

**Objectives:** To evaluate the frequency and distribution of pelvic nodes metastases, in intermediate-high risk prostate cancer (PCa) patients (pts), who underwent open radical prostatectomy (ORP) and superextended pelvic lymph node dissection (sePLND).

**Patients and Methods:** We retrospectively evaluated 630 consecutive pts with clinically localized, intermediate-high risk PCa, treated with ORP and sePLND from 2009 to 2016 at a single institution. The sePLND always removed all nodal/fibro-fatty tissue of the internal iliac, external iliac, obturator, common iliac, and presacral regions.

**Results:** Positive lymph nodes (LN+) were found in 133 pts (21.1%). The median number of removed nodes and LN+ was 25 and 1, respectively. LN+ were found in 64 (48.1%), 58 (43.6%), 53 (39.8%), 16 (12%), and 20 (15%) pts and were present as a single site in 27 (20.3%), 22 (16.5%), 20 (15%), 0, and 6 (4.5%) cases in the internal iliac, external iliac, obturator, common iliac, and presacral chain, respectively. An ePLND would have correctly staged 127 (95%) pts but removed all LN+ in only 97 (73%) pts. Presacral nodes harbored LN+ in 20 patients. Among them, 18 were high-risk patients. Moreover, all but 1 pts with common iliac LN+ were in high risk group.

**Conclusions:** These results suggest that removal of presacral and common iliac nodes could be omitted in intermediate risk pts. However, a PLND limited to external iliac, obturator, and internal iliac region may be adequate for nodal staging purpose, but not enough accurate if we aim to remove all possible site of LN+ in high risk pts.

## Introduction

The presence of nodal metastases (LN+) remains an adverse prognostic factor in patients treated for prostate cancer (PCa), and, in intermediate and high-risk patients (pts), current European Association of Urology (EAU) PCa guidelines recommend performing extended pelvic lymph node dissection (ePLND) in case of an estimated risk for LN+ >5% (1). Indeed, even though several different pre-treatment imaging techniques have been evaluated for nodal staging, their sensitivity and accuracy are still limited (2–6). Sentinel lymph node (SLN) detection has been proposed as a potential alternative to PLND. However, considering the complex drainage pattern of the prostate, and the low sensitivity of the technique for the detection of LN+, fluorescence SLN detection should not be considered, at present, an alternative to an accurate PLND in higher risk patients (7).

Therefore, PLND still remains the gold standard for nodal staging, and generally consensus has been reached on the need of a PLND extended at least to the obturator fossa, external and internal iliac vessels (1, 8). Moreover, Mattei et al. demonstrated, in their mapping study, that a LND extended up to the ureteric crossing would allow the removal of approximately 75% of all primary landing sites, while only 63% were located in the intrapelvic area (9). Recently, Joniau et al. suggested to add presacral node removal to ePLND, in order to remove LN+ in 97% of pts (10).

In our study, we aimed to describe and confirm the frequency and distribution of pelvic nodes metastases in intermediate-high risk prostate cancer (PCa) patients (pts), who underwent open radical prostatectomy (ORP) and super-extended pelvic lymph node dissection (sePLND), which adds common iliac and presacral nodes to an ePLND template.

## Patients and methods

The protocol for the research project was approved by our institutional Ethics Committee (registration number 2017/0164). Six-hundred and thirty consecutive pts with clinically localized, intermediate-high risk PCa, treated with ORP and sePLND from 2009 to 2016 at a single institution, were retrospectively analyzed. According to the EAU risk groups for biochemical recurrence of localized and locally advanced prostate cancer, 221 pts (35%) were pre-operatively included in the intermediate risk group (PSA 10–20 ng /mL or GS 7 or cT2b), and 409 pts (65%) were in the high-risk group (PSA > 20 ng / mL and/or GS > 7 and/or cT2c or higher clinical stage). All patients were staged by abdominal CT and bone scan, and were cN0 M0. All pts were treated with sePLND, however, nomograms were not used to calculate the risk of lymph node invasion.

Surgery was performed by three experienced surgeons. The sePLND always consisted of the removal of all nodal/fibro-fatty tissue at the following regions, according to the previous description of Joniau et al. (10):

- **Common iliac region**. From the internal/external iliac arteries bifurcation up to the ureteric crossing, from psoas muscle, and genitofemoral nerve laterally to the common iliac artery medially.
- **Presacral region**. Triangular region between medial borders of common iliac arteries and line connecting internal/external iliac arteries' bifurcations; dorsal border: promontory and proximal sacrum (S1–S2).
- **External iliac region**. From the bifurcation of internal/external iliac arteries to circumflex iliac vein, from psoas muscle, and genitofemoral nerve laterally, to the external iliac artery medially.
- **Obturator fossa region**. From the bifurcation of the internal/external iliac arteries to pelvic floor, obturator nerve, and medial border external iliac artery.
- **Internal iliac region**. From the bifurcation of internal/external iliac arteries to pelvic floor, bladder wall, obturator nerve.

Specimens from each anatomic region were sent in separate packets. Fatty tissue containing lymph nodes were fixed in 10% buffered formalin. The number of nodes was obtained from pathological records for each anatomic group. The macroscopic specimen assessment was based on tactile and visual criteria. Nodes larger than 2 cm were sampled in multiple blocks. If no LNs were macroscopically detected, all fat tissue was processed. All blocks were embedded in paraffin, cut at 3 μm, and stained with hematoxylin–eosin. A single dedicated uro-pathologist (D.C.) evaluated the presence of LN+. Lymph node metastasis was defined as nodal architecture totally or partially replaced by a nodular or diffused infiltrate of neoplastic prostatic cells. In a very few cases, immunohistochemical stain for cytokeratin (AE1/AE3; CAM5.2) was performed and multiple sections were analyzed for histologic presence of isolated tumor cells, to evaluate their epithelial origin. Specimens from radical prostatectomy were classified according to the 2010 TNM classification, and Gleason score was determined.

The primary endpoint of the study was the evaluation of frequency and distribution of nodal metastases. LN density (the number of LN+ divided by the total number of LNs removed) was also calculated for each region.

Moreover, the role of the extent of PLND in nodal staging and LN+ removal was evaluated. The removal of external iliac and of obturator nodes was considered as a limited PLND (lPLND); ePLND includes internal iliac nodes, while sePLND adds common iliac and presacral nodes to an ePLND template. We evaluated the concordance rate for nodal staging between a given PLND template and the super extended template, considered as reference for optimal staging (patient correctly staged). Furthermore, we assessed how many pts would have received a complete removal of positive nodes with lPLND and ePLND in comparison to sePLND results.

Complications were recorded at a minimum follow-up of 40 days as secondary endpoint. The five-grade modified Clavien system was retrospectively used to assess complications (11, 12). Complications related to LND were classified as follows lymphedema, symptomatic lymphoceles, deep venous thrombosis (DVT), pulmonary embolism, major vascular or ureteric injury, and sensory or motor neuropraxia. Pts were treated with LMWH (4000 IU of enoxaparin sodium s.c. injection daily) prophylaxis from the day of surgery for 4 weeks.

Means, medians, Interquartile ranges (IQR), and frequencies were used as descriptive statistics. All statistical tests were performed using SPSS software v 22 (IBM Corp., Somers, NY).

## Results

### Primary endpoint: frequency and distribution of nodal metastases

Positive nodes (LN+) were found in 133 patients (21.1%). Of those, 32 were in the intermediate-risk group (14.5%) and 101 in the high-risk group (25%). Patient characteristics of the overall population and of positive nodes patients are reported in Tables 1, 2.

**Table 1:** Patient characteristics (overall population), according to intermediate and high-risk group.

**Clinical variables**	**Intermediate risk pts (*n* = 221)**	**High risk pts (*n* = 409)**
**Age (years) Mean/Median (IQR)**	63/65 (61–70)	65/67 (61–72)
**PSA (ng/ml) Mean/Median (IQR)**	8.9/6.5 (5.1–11.9)	13.3/11.4 (7.1–17.6)
**Clinical T stage; (%)**		
1c	93 (42.1)	58 (14.2)
2a	60 (27.1)	34 (8.4)
2b	68 (30.8)	30 (7.3)
2c	–	86 (21.0)
3a	–	155 (37.9)
3b	–	45 (11.0)
4	–	1 (0.2)
**Gleason score at biopsy; (%)**		
3+4	133 (60.2)	118 (28.9)
4+3	88 (39.8)	108 (26.4)
8		115 (28.1)
9		66 (16.1)
10		2 (0.5)
**PATHOLOGIC VARIABLES**		
**pT stage; (%)**		
2c	110 (49.8)	149 (36.4)
3a	67 (30.3)	123 (30.1)
3b	44 (19.9)	135 (33.0)
4	–	2 (0.5)
**Pathologic Gleason score; (%)**		
3+4	88 (39.8)	101 (24.7)
4+3	77 (34.8)	85 (20.8)
8	39 (17.7)	76 (18.6)
9	17 (7.7)	145 (35.5)
10	–	2 (0.5)
**# of LNs removed Mean/Median (IQR)**	21/20 (14–25)	23/21 (15–31)
**# of patients with LN**+ **(%)**	32 (14.5)	101 (25)

**Table 2:** Positive nodes patient characteristics, according to intermediate and high-risk group.

**Clinical Variables**	**Intermediate risk pts (*n* = 32)**	**High risk pts (*n* = 101)**
**Age (years) Mean/Median (IQR)**	66/67 (62–70)	66.7/67 (62–72)
**PSA (ng/ml) Mean/Median (IQR)**	9.3/7.1 (5.30–12.6)	15.1/13.0 (7.9–18.4)
**Clinical T stage; (%)**		
1c	13 (40.6)	3 (2.9)
2a	10 (31.2)	5 (4.9)
2b	9 (28.2)	2 (2.0)
2c	–	19 (18.8)
3a	–	50 (49.5)
3b	–	21 (20.8)
4	–	1 (1.0)
**Gleason score at biopsy; (%)**		
3+4	10 (31.2)	3 (2.9)
4+3	22 (68.8)	42 (41.6)
8		34 (33.7)
9		20 (19.8)
10		2 (2.0)
**PATHOLOGIC VARIABLES**		
**pT stage; (%)**		
2c	9 (28.1)	8 (7.9)
3a	10 (31.2)	22 (21.8)
3b	13 (40.7)	70 (69.3)
4	–	1 (1.0)
**Pathologic Gleason score; (%)**		
3+4	5 (15.6)	2 (2.0)
4+3	11 (34.4)	41 (40.6)
8	8 (25.0)	28 (27.7)
9	8 (25.0)	28 (27.7)
10	–	2 (2.0)
**# of LNs removed Mean/Median (IQR)**	20/20 (14–27)	24/21 (16–30)
**# of positive LNs Mean/Median (IQR)**	2.4/1 (1–3)	2.6/1.5 (1–3)
**LN** + **distribution; # (%)**		
Internal iliac	14 (43.8)	50 (49.5%)
External iliac	13 (40.6)	45 (44.6%)
Obturator	11 (34.3)	42 (41.6%)
Common iliac	1 (3.1)	15 (14.8%)
Presacral	2 (6.2)	18 (17.8%)

The median number of removed nodes was 23 (IQR 16–27). The mean and median number of positive nodes were 2.6 and 1 (IQR: 1–3), respectively. The median number of removed nodes was 5, 6, 8, 2, and 1 for internal iliac, external iliac, obturator, common iliac, and presacral site, respectively.

**Figure 1:**
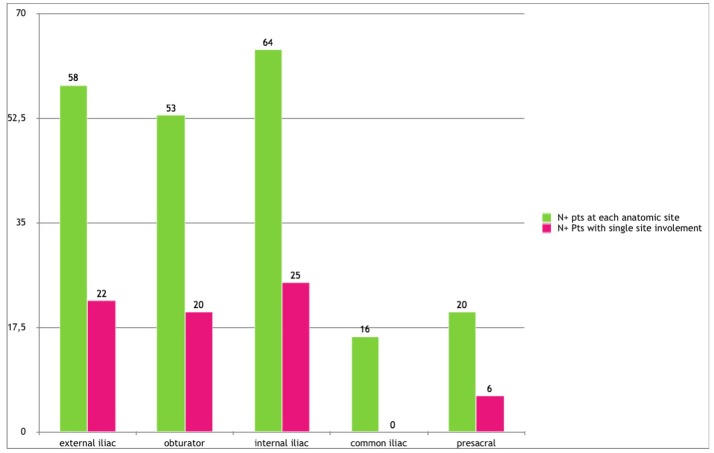
Lymph node metastases distribution.

Out of the 133 Pts, nodal metastases were found in 64 (48.1%), 58 (43.6%), 53 (39.8%), 16 (12%), and 20 (15%) pts in the internal iliac, external iliac, obturator, common iliac, and presacral sites, respectively. However, when we analyzed the presence of LN+ only in a single anatomic area, nodal metastases were present in 27 (20.3%), 22 (16.5%), 20 (15%), 0, and 6 (4.5%) pts in the internal iliac, external iliac, obturator, common iliac, and presacral sites, respectively (Figure 1). Metastases at common iliac nodes were always associated with concomitant involvement of external iliac, obturator and/or internal iliac nodes. Of interest, presacral nodes harbored LN+ in 20 patients. Among them, 18 were high-risk patients. Furthermore, all but 1 pts with common iliac LN+ were in high risk group.

**Figure 2:**
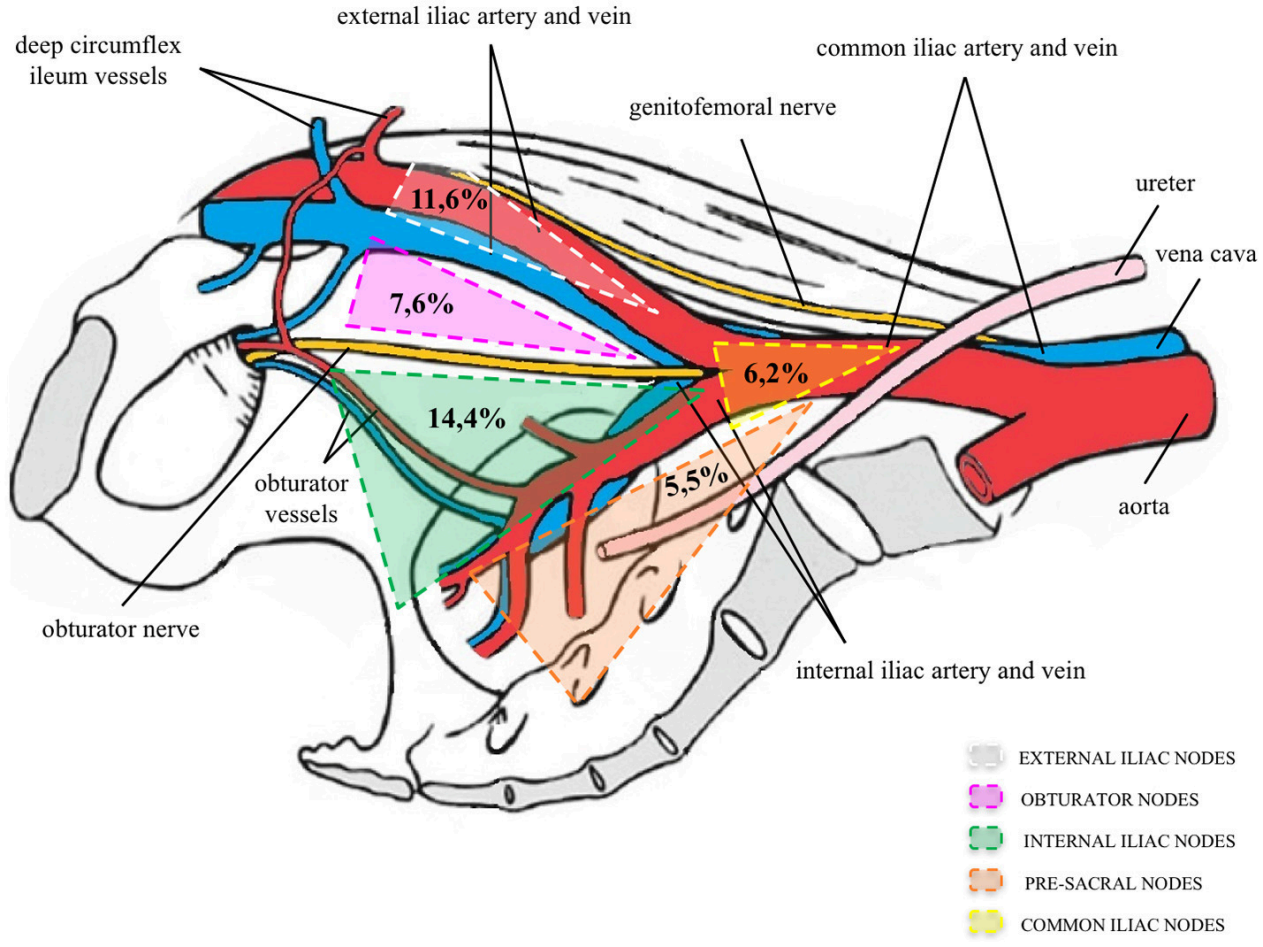
Percentage of positive nodes of the total number of lymph nodes removed per region. Modified with permission from Elsevier (9).

In LN+ pts, a total of 3,201 LNs were removed, and 411 LNs were positive (mean LN density 12.8%; median 8.9%; IQR 4.7–15.8%). LN density was also calculated for each nodal region, to confirm a hierarchic order in nodal metastases distribution. Mean LN density was 14.4, 11.6, 7.6, 6.2, and 5.5% for internal iliac, external iliac, obturator, common iliac, and presacral regions, respectively (Figure 2). Moreover, of 411 positive LNs, 129 (31.4%) were detected in the internal iliac region, 121 (29.5%) in the external iliac region, while 98 LNs (23.9%) were found in the obturator, 36 (8.9%) in the common iliac, and 27 (6.6%) in the presacral regions, respectively.

A lPLND would have correctly staged 102 (77%) pts and would have removed all LN+ in 37 (28%) Pts. An ePLND would have correctly staged 127 (95%) pts but removed all LN+ in only 97 (73%) pts.

### Secondary endpoint: complication related to PLND

Data are available for 470 pts out of 630 pts. Ninety-four of 470 pts (20%) had post-operative complications related to PLND. Fifty-two pts (11%) had lymphedema, which was transient in 43 cases. Fourty-eight patients (10%) developed a lymphocele, requiring percutaneous drainage in 23 cases (4.9%; Clavien IIIa). In 3 pts, intraoperative lesion of hypogastric vein, requiring intra and post-operative blood transfusion (Clavien II), was recorded. In one case, section of the ureter required termino-terminal anastomosis and DJ placement; subsequently the patient experienced ureteral stenosis and underwent endoscopic ureteral balloon dilatation and DJ stenting (Clavien IIIb). Five pts experienced transient neuropraxia of the obturator nerve, while three pts presented with DVT (one of those with asymptomatic pulmonary embolism) and required anticoagulant therapy.

## Discussion

Evaluation of nodal involvement in PCa is still a challenging issue. Despite several pre-operative imaging tecnique and SLN detection have been tested for nodal staging, PLND remains, at present, the most accurate procedure for patients with intermediate and high risk PCa (1). There is agreement that, in these pts, PLND dissection should be extended and, at the same time, that the extent of the PLND should never be based on the number of resected nodes (13). Indeed, the number of nodes retrieved varies among patients, according to how the specimen were handled by pathologists or the method by which the LN specimens were submitted to pathology (14). The extent of PLND should be anatomically defined: the template should include at least removal of all lymphatic tissue in the obturator fossa, along the hypogastric, and the external iliac vessels, bilaterally (1, 8).

Recently, a retrospective single-surgeon series investigate the frequency and distribution of nodal metastases in 427 patients treated with radical prostatectomy for localized PCa (15). Positive nodes were detected in the external iliac region only in 37% of the pts. On the contrary, 60 and 49% of pts had at least 1 LN+ in the obturator and hypogastric site, respectively.

However, whether PLND should be extended to other additional lymphatic regions is matter of debate and requires an adequate balance between the advantage of LN+ yield and the risk of potential morbidity.

As a primary endpoint, our study evaluated the frequency and distribution of nodal metastases. Out of the 133 pts, nodal metastases were found in 64 (48.1%), 58 (43.6%), and 53 (39.8%) pts, in the internal iliac, external iliac, and obturator sites. These results seem to indicate that PCa metastases preferentially disseminated to internal iliac nodes. Joniau et al. first described a predilection for the internal iliac region by analyzing the relationship between the number of affected and resected LNs. The predominant site for LN+ was the internal iliac region (35%), followed by the external iliac region (26%) and the obturator fossa region (*n* = 23, 25%). According with their findings, our data supported a hierarchic distribution of nodal metastases in the drainage chains of the internal iliac region (31.4%), followed by the external iliac region (29.5%) and the obturator fossa region (23.9%). Moreover, in our population, LN density was 14.4, 11.6, 7.6, 6.2, and 5.5% for internal iliac, external iliac, obturator, common iliac, and presacral regions, respectively.

Nonetheless, roughly a 25% of LN+ was detected in common iliac (16 pts; 12%) and presacral sites (20 pts, 15%), respectively. Similar results were described by Gandaglia et al. (16), who recently showed that 19% of high-risk PCa pts harbored LN+ in common iliac or presacral region. These results are also in agreement with the mapping study of Mattei et al. (9). The Authors highlighted that only 63% of primary lymphatic landing sites were located in the region of ePLND, while another 16% are found along the common iliac vessels, and 8% in the presacral/pararectal regions. Skipping these regions during PLND would result in a significant percentage of nodal metastases missed, at least in high-risk pts.

Moreover, when we analyzed the presence of LN+ only in a single anatomic area, nodal metastases were present in 27 (20.3%), 22 (16.5%), 20 (15%), 0, and 6 (4.5%) pts, in the internal iliac, external iliac, obturator, common iliac, and presacral region, respectively. Instead, no metastases at common iliac nodes were detected in the absence of lower pelvic nodes involvement, and no skip lesions were found between the lower pelvic and common iliac regions.

A previous mapping study using SPECT imaging after intra-prostatic injection of Tc-99m nanocolloid demonstrated that common iliac nodal involvement is always associated with concomitant LN invasion in either the external or the internal iliac sites, as was in our study (17). In a prospective mapping study evaluating a group of 19 very high-risk patients, Briganti et al. (18) showed that all patients with LN+ in the common iliac region also had positive lower pelvic nodes. Moreover, retroperitoneal LN+ were detected only when the common iliac nodes were also involved. Our results confirm these previous findings, and consolidate the theory of the ascending pathway of PCa nodal metastases, up to the retroperitoneal chains from lower pelvic areas through the common iliac nodes. On the contrary, recent publication (19) showed that 22.7% of positive LNs were found in the common iliac area, and three patients (12.5%, 3/24) had positive LNs exclusively in this area without intrapelvic involvement.

Finally, we evaluated the role of the extent of PLND in nodal staging and LN+ removal. Compared to sePLND, a lPLND would have correctly staged 102 (77%) pts and would have removed all LN+ in only 37 (28%) pts. An ePLND would have correctly staged 127 (95%) pts but removed all LN+ in only 97 (73%) pts. Similar results were demonstrated in the paper of Joniau et al. (10). Moreover, in our study, presacral nodes harbored LN+ in 20 patients. Among them, 18 were high-risk patients. Furthermore, common iliac LN+ were nearly exclusively found in high risk pts. These results suggest that a PLND limited to external iliac, obturator, and internal iliac region should be enough to obtain an adequate nodal staging in pts at intermediate-high risk of biochemical recurrence. However, in order to achieve a complete removal of LN+, presacral and common iliac chains should also be dissected in high risk pts, while in those at intermediate risk could be omitted. Nonetheless, considering the well-known lack of accuracy of current preoperative staging and grading, the choice of performing an ePLND or sePLND may be influenced by different risk factors present within the intermediate risk group patients. A preoperative risk assessment of LN+ could be helpful to decide the extent of PLND according to individual patient characteristics. Recently, Gandaglia et al. (16) showed that, according to Briganti nomogram, LN+ at common iliac and presacral regions were found in <5% of patients with an LNI risk of <30%. They concluded that a sePLND including these regions should be performed only in men with a risk of LNI ≥30%, in order to avoid potential side effects in pts less likely to harbor LN+, while reducing the risk of under-staging men at higher risk of pelvic nodal metastes.

Moreover, SLN detection has also been investigated as a potential alternative to PLND. Particularly, the use of the fluorescent dye indocyanine green (ICG) has recently been explored in PCa surgery (6). However, a recent study suggests that, due to the complex drainage pattern of the prostate, and the low sensitivity of the technique for the detection of nodal metastases, fluorescence SLN detection is not enough reliable, at present, to be considered as an alternative to an accurate PLND in higher risk patients (7).

As a secondary end-point, we evaluated the complications related to PLND. Indeed, many studies showed that PLND and its extent are associated with worse intraoperative and perioperative outcomes. Out of 470 pts with positive nodes detected at sePLND, we reported a 20% of intraoperative and post-operative complications related to sePLND, the majority of those being lymphoceles, that require in approximately half of cases percutaneous drainage. These results are similar to those previously reported in case of extended or sePLND (10, 18, 20). Even though the incidence of complications seems to be higher than in case of standard PLND (21, 22), no significant differences were observed in other retrospective studies between ePLND compared to limited or standard PLND (23, 24). However, it can be highlighted that the incidence of life-threatening complications, such as massive bleeding or DVT and/or pulmonary embolism, are rare, but not negligible. For this reason, any effort must be done to better tailor the extent of PLND to the individual patient risk.

It can also be argued that the therapeutic role of PLND during radical prostatectomy is controversial: a recent systematic review (25) concluded that a direct therapeutic effect is still not evident from the current literature. Nevertheless, many retrospective studies suggested direct curative effect in pts with limited nodal involvement that is entirely removed at the time of surgery (26), and reported direct correlation with the removal of a higher number of nodes (27). Furthermore, an indirect effect of PLND may consist in the identification of pts who may benefit from adjuvant treatments, thus improving survival outcomes (28).

This study has some limitations. First, the power of our findings may be somewhat limited by the retrospective nature of the study and by the presence of multiple surgeons, who performed the PLND. However, these 3 surgeons applied a highly standardized super-extended template in intermediate-high risk patients, according to our Institution indications. Second, the rate of pT3b seems higher than in other casistics, which suggest possible patient selection; however, our study considered all the consecutive pts treated from 2009 to 2016, and pathological features are similar to those shown by other recent papers (Table 3). Third, common iliac nodes were dissected up to the ureteric crossing, while we have no pathologic information on LNs above the ureteric crossing, at the aortic bifurcation or higher. According to the data of Briganti et al, we can presume that higher location of LN+ would have been found only in those pts with common iliac involvement. Finally, due to the retrospective nature of the study, data on presence of LN+ in the periprostatic fat are not available. Recently, several studies based on robotic surgery, reported that approximately 10–15% of patients had LNs in the periprostatic area and LN+ were found in this area in case of multiple metastases, as well as a single region of nodal involvement (18, 31).

**Table 3:** Baseline characteristics of studies evaluating the role of sePLND.

**Study**	**PLND**	**Number of patients**	**Age at surgery (IQR)**	**Initial PSA**	**Biopsy histology *N* (%)**	**Clinical T Stage *N* (%)**	**Pathological T Stage *N* (%)**	**Number of LN dissected**	**Number of positive LN**
**Gandaglia et al. (16)**	Super-extended	471	66.7 IQR (60.9–71.6)	10.0 IQR (6.2–25.4)	**grade group** **1** 86 (18.3) **2** 70 (14.9) **3** 54 (11.5) **4** 146 (31.0) **5** 115 (24.4)	≥cT3	**pT2** 163 (34.6) **pT3a** 123 (26.1) **pT3b** 161 (34.2) **pT4** 24 (5.1)	23 (IQR 18–30)	3 (IQR 2–9)
**Joniau et al. (10)**	Super-extended	74	64.5 IQR (42.9–73.9)	10.4 IQR (1.5–70.9)	**Gleason score** **6** 1 (1.6) **7 (3**+**4)** 23 (31.1) **7 (4**+**3)** 17 (23) **8** 21 (28.4) **9 (4**+**5)** 7 (9.5) **9 (5**+**4)** 2 (2.7) **10** 1 (1.6)	**T1c** 1(1.6) **T2 a** 2(2.7) **T2b** 3(4.1) **T2c** 14(18.9) **T3a** 42 (56.8) **T3b** 11 (14.9) **T4** 1(1.6)	**pT2b** 2 (2.7) **pT2c** 30 (40.5) **pT3a** 20 (27) **pT3b** 19 (25.7) **pT4** 3 (4.1)	21 (IQR 7–49)	2 (IQR 1–3)
**Mattei et al. (9)**	Super-extended	34	63 (R:51–72)	8 (R:0.3–40)	NR	cT1 or cT2	NR	26 (R:13–44)	NR
**Heidenreich et al. (20)**	Standard	100	63.5 (R:49–72)	14.9 (R:1.6–109)	**Gleason score** 5.2 ±2.6 (SD)	**T1** 10 (10%) **T2** 65 (65%) **T3** 25 (25%)	3.5 (R: 1–4)	11 (R: 6–19)	12% (*n* = 12)
	Super-extended	103	61.8 (R: 51–71)	15.9 (R:1.2–129)	**Gleason score** 4.6 ±2.3 (SD)	**T1** 9 (8.7%) **T2** 61 (61%) **T3** 33 (33%)	3.6 (R: 1–4)	28 (R: 21–42)	26.2% (*n* = 27)
**Kim et al. (22)**	Standard	294	65 (R: 60–69)	8.4 (R:5.3–37.7)	**Gleason score** **≤** **6** 98 (33.4) **7** 142(48.3) **8–10** 54 (18.3)	**T1** 195 (66.3) **T2** 63 (21.4) **T3** 36 (12.3)	**T2** 183 (62.2) **T3a** 84 (28.6) **T3b** 27 (9.2)	12 (R:9–16)	3.4% (*n* = 10)
	Extended + common iliac	170			**Gleason score** **≤** **6** 30 (17.7) **7** 67 (39.4) **8–10** 73 (42.9)	**T1** 78 (45.9) **T2** 70 (41.2) **T3** 22 (12.9)	**T2** 96 (56.5) **T3a** 49 (28.8) **T3b** 25 (14.7)	21 (R:16–25)	13.5% (*n* = 23)
**Eden et al. (29)**	Standard	311	63 (43–76)	11 (2–20)	**Gleason score** 7 (4–10)	**T1** 102 (32.8) **T2** 196 (63.0) **T3** 13 (4.2)	**NR**	6.1 (2–8)	
	Extended + common iliac	121	63 (43–74)	8 (1–15)	**Gleason score** 7 (6–10)	**T1** 40 (33.1) **T2** 174 (57.0) **T3** 12 (9.9)	**NR**	17.5 (2–23)	
**Naselli et al., (30)**	Limited	98	NR	6.43 (R:1.96–65)	**NR**	**NR**	<**T3** 73 (74.5) **T3a** 22 (22.4) **T3b** 8 (8.2)	6 (R:2–14)	1% (*n* = 2)
	Extended + common iliac	249	NR	7.22 (R:2.2–98)	**NR**	**NR**	<**T3** 168 (67.5) **T3a** 65 (30.1) **T3b** 39 (15.7)	16 (R:10–67)	11.7% (*n* = 29)
**Yuh et al. (23)**	Limited	204	64 (IQR:58–70)	5.9 (IQR:4.4–91)	**Gleason score** **6** 13 (6.4) **3**+**4** 112 (54.9) **4**+**3** 45 (22.1) **8** 25 (12.2) **9** 9 (4.4)	**T1** 147 (72.1) **T2** 56 (27.4) **T3** 1 (0.5)	**T2a/b** 15 (7.4) **T2c** 118 (57.8) **T3a** 48 (23.5) **T3b** 23 (11.3)	7 (IQR:5–9)	3.9% (*n* = 8)
	Extended + common iliac	202	64 (IQR:58–89)	5.5 (IQR:4.2–8.3)	**Gleason score** **6** 12 (5.9) **3**+**4** 112 (59.9) **4**+**3** 40 (19.8) **8** 23 (11.4) **9** 6 (3.0)	**T1** 139(68.8%) **T2** 61 (30.2) **T3** 2 (1.0)	**T2a/b** 25 (12.4) **T2c** 122 (60.7) **T3a** 34 (16.8) **T3b** 21 (10.4)	21.5 (IQR 17–27)	11.9 (*n* = 24)

*sPLND, standard pelvic lymph node dissection; ePLND, extended pelvic lymph node dissection; LN, lymph nodes; PSA, prostate-specific antigen; SD, standard deviation; R, range; IQR, interquartile range; NR, not reported*.

In conclusion, nodal metastases were found predominantly in the template of an ePLND. However, it should be noted that roughly a 25% of LN+ was detected in common iliac and presacral sites. Moreover, LN+ were present only in internal iliac or presacral regions in 24% of cases. On the contrary, metastases at common iliac nodes were always associated with concomitant involvement of lower pelvic chains, confirming the theory of nodal metastases ascending pathway. An ePLND would have correctly staged 127 (95%) pts but would have removed all LN+ in only 97 (73%) pts. Moreover, in our study, common iliac LN+ were nearly exclusively found in high risk pts, and, in case of positivity in the presacral area, the majority of the patients were high risk.

These results suggest that removal of presacral and common iliac nodes could be omitted in pts at intermediate risk group.

However, a PLND limited to external iliac, obturator and internal iliac region may be adequate for nodal staging purpose, but not enough accurate if we aim to remove all possible site of LN+ in high risk pts.

## Author contributions

MR developed the project, analyzed the data, and wrote the manuscript. MN developed the project, collected the data, and edited the manuscript. GL analyzed the data. FP collected the data. MS developed the project and collected the data. AS wrote the manuscript. DA collected the data. DC collected the data and edited the manuscript. LD developed the project and edited the manuscript.

### Conflict of interest statement

The authors declare that the research was conducted in the absence of any commercial or financial relationships that could be construed as a potential conflict of interest. The handling Editor declared a past co-authorship with the authors MR and LD.
